# DNA Metabarcoding Reveals Diet Overlap between the Endangered Walia Ibex and Domestic Goats - Implications for Conservation

**DOI:** 10.1371/journal.pone.0159133

**Published:** 2016-07-14

**Authors:** Berihun Gebremedhin, Øystein Flagstad, Afework Bekele, Desalegn Chala, Vegar Bakkestuen, Sanne Boessenkool, Magnus Popp, Galina Gussarova, Audun Schrøder-Nielsen, Sileshi Nemomissa, Christian Brochmann, Nils Chr. Stenseth, Laura S. Epp

**Affiliations:** 1 Centre for Ecological and Evolutionary Synthesis (CEES), Department of Biosciences, University of Oslo, Oslo, Norway; 2 Norwegian Institute of Nature Research, Sluppen, Trondheim, Norway; 3 Department of Zoological Science, Addis Ababa University, Addis Ababa, Ethiopia; 4 Natural History Museum, University of Oslo, Oslo, Norway; 5 Department of Plant Biology and Biodiversity Management, Addis Ababa University, Addis Ababa, Ethiopia; 6 Alfred Wegener Institute, Helmholtz Centre for Polar and Marine Research, Periglacial Research, Potsdam, Germany; University of Sassari, ITALY

## Abstract

Human population expansion and associated degradation of the habitat of many wildlife species cause loss of biodiversity and species extinctions. The small Simen Mountains National Park in Ethiopia is one of the last strongholds for the preservation of a number of afro-alpine mammals, plants and birds, and it is home to the rare endemic Walia ibex, *Capra walie*. The narrow distribution range of this species as well as potential competition for resources with livestock, especially with domestic goat, *Capra hircus*, may compromise its future survival. Based on a curated afro-alpine taxonomic reference library constructed for plant taxon identification, we investigated the diet of the Walia ibex and addressed the dietary overlap with domestic goat using DNA metabarcoding of faecal samples. Faeces of both species were collected from different localities in the National Park. We show that both species are browsers, with forbs, shrubs and trees comprising the largest proportion of their diet, supplemented by grasses. There was a considerable overlap in dietary preferences. Several of the preferred diet items of the Walia ibex (*Alchemilla* sp., *Hypericum revolutum*, *Erica arborea* and *Rumex* sp.) were also among the most preferred diet items of the domestic goat. These results indicate that there is potential for competition between the two species, especially during the dry season, when resources are limited. Our findings, in combination with the expected increase in domestic herbivores, suggest that management plans should consider the potential threat posed by domestic goats to ensure future survival of the endangered Walia ibex.

## Introduction

Human population expansion throughout the world causes significant loss of biodiversity, including species extinctions [1]. One of the important factors that may threaten the survival of endangered species is competition for resources between livestock and wildlife. Several studies on the interactions between livestock and native wildlife species have demonstrated a negative impact on the native population. In the cold deserts of the trans-Himalayas, domestic sheep and goats impose resource limitations for the Himalayan ibex (*Capra sibirica*), leading to an exclusion of the native ungulate from its optimal habitat [2]. The Iberian ibex (*Capra pyrenaica*) was also displaced to suboptimal habitats in the presence of extensive goat livestock in central Spain [3]. In the Italian Alps, the Alpine chamois (*Rupicapra rupicapra*) has moved upslope into an entirely novel altitudinal range in the presence of domestic sheep, with an almost 50% decrease in the availability of suitable foraging habitat as a consequence [4]. The presence of domestic goats (*Capra hircus*) in particular can have devastating effects on the plant community, as has been shown on several Pacific islands where consumption and trampling of native plants has sometimes led to transformation of the entire ecosystem structure [5, 6].

**Fig 1:**
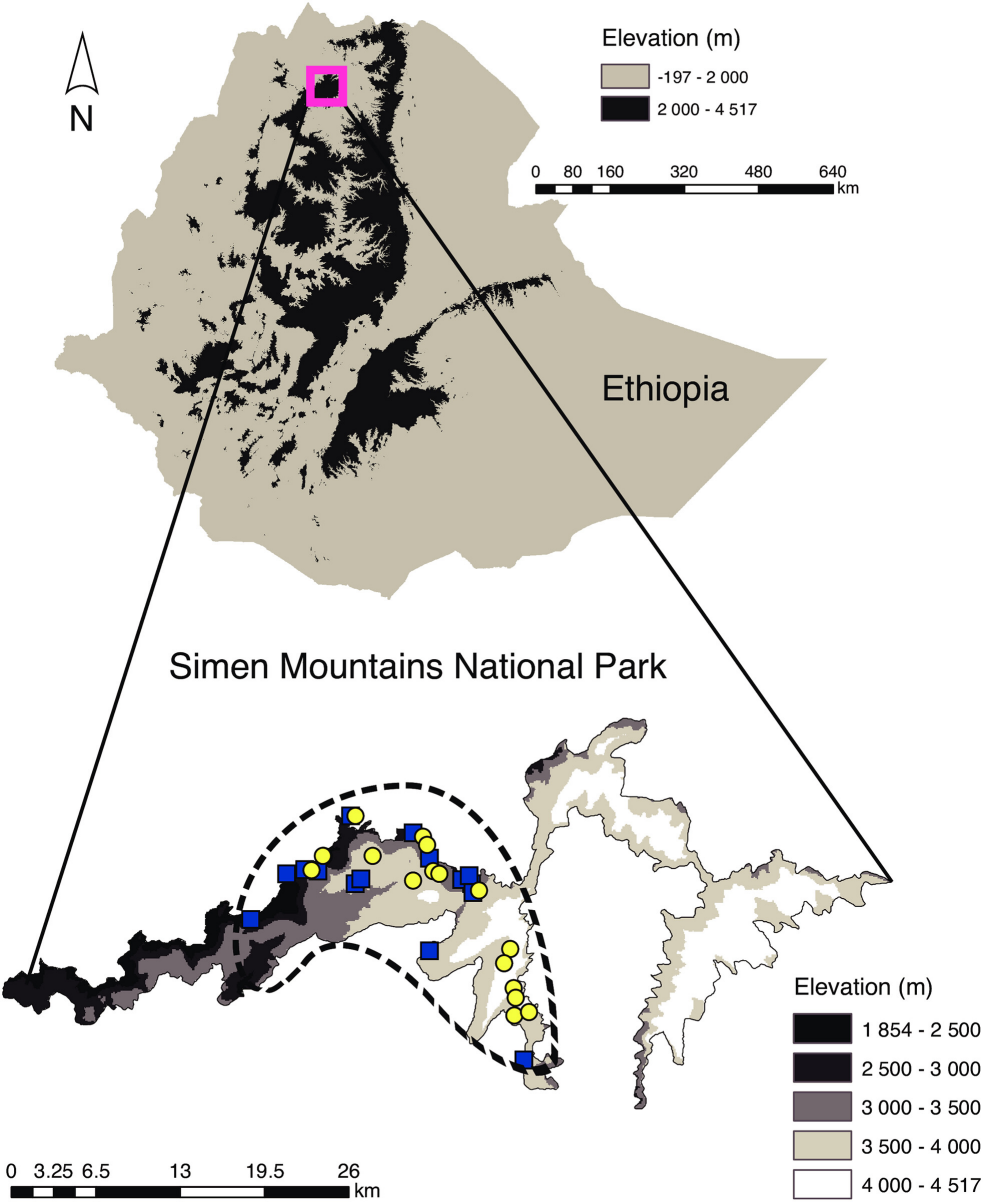
Map of Ethiopia and the Simen Mountains National Park. Collection sites of the individual samples are shown (yellow circles: Walia ibex; blue squares: domestic goat). The dashed line indicates the main habitat range of the Walia ibex.

Tropical mountain ecosystems are biodiversity hotspots with significant risks associated with land use, land cover change and global warming [7]. The Ethiopian mountains are one of these hotspots, and they are home to several unique large mammal species, such as the Walia ibex (*Capra walie*) and the Ethiopian wolf (*Canis simensis*). Their numbers have been reduced due to habitat loss and fragmentation of suitable habitats. With the increase of the human population and an intensification of agriculture [8, 9], this degradation of the highland ecosystem will potentially lead to further population fragmentation and local extinction [10]. The Simen Mountains National Park in Ethiopia (Fig 1) is one of the last strongholds for the preservation of a diversity of afro-alpine mammals, plants and birds. Given its small size, the park is highly vulnerable to increased modification by anthropogenic activities and has been listed as a World Heritage Site in Danger [11]. Over the years, the area of cultivated land has increased and hence the availability of natural alpine vegetation has been reduced [11]. Moreover, the abundance and distribution of livestock has increased, resulting in a substantial proportion of eroded and heavily overgrazed grasslands in the National Park [12, 13]. Settlement, development of infrastructure and semi-urbanization of rural areas in the Simen mountains have also intensified [13] and environmental degradation has resulted in population declines of several flagship species.

The Walia ibex is a rare endangered species endemic to the Simen Mountains National Park and has a very narrow distribution range (Fig 1, see also [14]). It inhabits plateaus, gorges and escarpments ranging from 2700 to 4430 m in altitude. The species occurs as a single, small population with very low genetic diversity [15]. In 1994 the population size was estimated as low as 150 [11], and the species was subsequently classified as critically endangered in 1996 [16]. Concerted conservation efforts have led to an apparent increase in population size to almost 750 individuals [17], and the species status has been upgraded to endangered [16]. Land use in the native region of the Walia ibex is characterized by mixed crop-livestock farming [13], with goats and sheep among the most common livestock. The impact of overgrazing has previously been documented in the Simen mountains [12], where livestock may forage year-round in areas as high as 4250 m. In 2007 it was estimated that about 77 000 small ruminants were found in villages and settlements within and around the national park, of which 23% are goats [18]. This means that large numbers of domestic goat graze freely within and around the Simen Mountains National Park, and during the dry season 95.3% of the protected area has been documented to be affected by goats, compared to 42.2% during the rainy season [19]. Given the small distribution range of the Walia ibex in its restricted mountain ecosystem, the presence of a large number of domestic goats may pose a serious threat that can directly affect the survival of the population. Apart from resource competition between the two species, potential threats include hybridization [20] and transmission of parasites [21], but an actual assessment of the threats is lacking to date. Currently, there is no thorough understanding of the diet of the Walia ibex, and no assessment has been made of the level of dietary overlap between the Walia ibex and its most likely competitor, the domestic goat. The few studies conducted to date on the diet of the Walia ibex have relied on direct observation of foraging [14, 22], and have not been conclusive. Such observational studies are difficult and time consuming, especially for species living in poorly accessible areas, and require considerable training of the observer to be able to identify food items [23].

In recent years, DNA metabarcoding, an approach that combines PCR amplification of a standardized, short marker for species identification with next generation sequencing technology, has been applied as a novel tool in diet studies [24], both using gut contents [25, 26] and faecal samples [25]. DNA metabarcoding has proven useful for small and medium-sized mammals [25, 27–30] as well as for larger herbivores [31, 32]. In this study, we investigated the diet of the Walia ibex living in the Simen Mountains National Park using DNA metabarcoding to identify food plants from faeces, and compared it to data retrieved for the domestic goat. Taxonomic assignment of the DNA sequences retrieved from faeces was achieved using a new, extended version of a curated afro-alpine taxonomic reference library (version 2.0, see [33] for version 1.0) constructed by sequencing DNA from taxonomically verified specimens of most afro-alpine plant species (S1 Table). Specifically, we assessed the diet overlap between the two herbivore species and evaluated the results in a conservation context.

## Methods

### Study area and sample collection

The study was conducted in the Simen Mountains National Park in northern Ethiopia (Fig 1). The park is the only natural UNESCO World Heritage Site in Ethiopia, covering an area of 412 km^2^. Most of the park is mountainous, with elevations ranging from 1900 to 4543 m, and it hosts a range of alpine and subalpine habitats and species. The three main vegetation belts described for afro-alpine ecosystems and surroundings in tropical Africa—the montane forest belt, the transitional ericaceous belt, and the uppermost afro-alpine belt—are typical features of the park [34]. The rainfall pattern in the Simen Mountains is characterized by a single rainy season with high amounts of rainfall occurring between June and September and an annual average rainfall of 1467 mm. December to April constitute the main dry months of the year, although dryness may extend to May. The mean annual temperature is 12°C [35], ranging between -2°C and +18°C, and snow occurs occasionally at altitudes over 3800 m.a.s.l. [36]. Weather conditions at the time of sampling were typical for the area and time of year. Wild and domestic ungulates occur in the national park, with Walia ibex (*Capra walie)*, klipspringer (*Oreotragus oreotragus*), common bushbuck (*Tragelaphus scriptus*) and grey duiker (*Sylvicapra grimmia*) as characteristic wild herbivore mammals [22]. Agriculture is the dominant land use system, and domestic animals—most commonly sheep and goats, but also cattle, horses, mules and donkeys—are present. A recent dry season census (2012) suggests that their numbers have reached about 300 000 head [37].

For our collection of faecal samples, we focused on the main habitat range of the Walia ibex (Fig 1). The species inhabits areas close to the edge of the escarpment, which protects them from predators and human disturbance [14]. Sample collection was restricted to areas frequently used by the species and shared with domestic goat, and we collected samples from different groups foraging along the mountain escarpments approximately 2 km apart from each other. We followed groups of animals and waited until they moved to avoid disturbing them. Foraging sites were checked for faeces soon after animals moved to other sites and samples were kept in vials with silica gel until arrival at the laboratory. Samples were collected in the dry season between March and May 2011 and locations of samples and their habitat were recorded (Fig 1). To minimize the probability for multiple samples from the same individual, we collected only one faecal sample from each encountered ibex group. Goat faeces were collected from browsing individuals found during the field survey and/or from nearby settlements where goats are confirmed to browse close to habitats of the Walia ibex.

### Ethics Statement

Our research was non-invasive and did not involve any capture or disturbance of animals. We only collected samples of plants and faeces for metabarcoding analyses. Access to the protected area was under permission from the Ethiopian Wildlife Conservation Authority (EWCA), a government institution that is responsible for the management and conservation of protected areas and wildlife and utilization of wildlife trophies and products in Ethiopia.

### Genetic analyses of faecal samples

DNA extraction from 48 faecal samples of assumed Walia ibex and domestic goat origin was performed at the Natural History Museum, University of Oslo, in a dedicated laboratory for analyses of DNA from samples with low DNA content. Approximately 20 mg of the inner part of the dried faecal pellets were used in each extraction, carried out according to the protocol described in [25]. Extraction blanks were included in each extraction round to monitor possible contamination.

To verify herbivore species identity we amplified and sequenced a 700 bp fragment of the *cytochrome b* (*Cyt-b*) gene as described in Pedrosa *et al*. [38]. In cases where the amplification of this fragment did not work, we amplified a shorter fragment of 426 bp [39]. PCR reactions were performed in 25 μl volumes containing 1.25 U Platinum^®^ Taq High Fidelity DNA Polymerase (Invitrogen), 1x PCR buffer, 2 mM MgSO4, 1 mM dNTPs, 0.4 mM of each primer, 0.8 mg/ml Bovine Serum Albumin (BSA) and 2.5 μl DNA extract. The PCR products were sequenced on an ABI 3730 sequencer, and downstream diet analyses were conducted for the samples identified as the target species from BLAST searches in GenBank (n = 23 for Walia ibex; n = 16 for domestic goat).

Identification of diet plants was performed using sequences of the P6 loop region of the *trn*L (UAA) intron, amplified with the universal primers *trn*L-g and *trn*L-h [40]. The primers were designed as fusion primers carrying the Roche 454 Lib-L adapters and Roche MID tags on the *g* primer to allow pooling of multiple samples for sequencing. DNA amplification of plants from the faecal extractions was carried out in a final volume of 25 μL, using 2.5 μL of DNA extract as template. The amplification mixture contained 1 U of AmpliTaq^®^ Gold DNA Polymerase (Applied Biosystems, Foster City, CA), 10 mM Tris-HCl, 50 mM KCl, 2.5 mM of MgCl_2_, 0.8 mg of bovine serum albumin, 0.25 mM of each dNTP and 0.2 μM of each primer. The mixture was denatured at 95°C for 10 min, followed by 35 cycles of 30 s at 95°C, 30 s at 50°C and 30 s at 72°C and a final 10 min elongation step at 72°C. For each extraction, three positive PCR products (verified on an agarose gel) were combined to minimize bias introduced in the PCR. These pooled PCR products were purified using Agencourt AMPure XP (Beckman Coulter) with a PCR product to bead ratio of 1:1, and subsequently quantified using a Qubit^®^2.0 Fluorometer (Invitrogen). The purified PCR products were pooled, taking into account the established DNA concentrations to obtain roughly equimolar amounts of DNA for each sample, and sequenced on half a plate of a Roche 454 GS FLX Titanium sequencing platform (Beckman Coulter Genomics).

Filtering of the sequences and taxonomic inference of molecular operational taxonomic units (MOTUs, see [41]) were performed using the OBITools package [42]. Filtering was performed as in [43], with an additional cleaning step using the program *obiclean* (part of the OBITools package, see [44]). Initial taxonomic annotation was carried out with the program *ecoTag* using both a local taxonomic reference library covering the afro-alpine flora including the flora of the habitat range of the Walia ibex (the Afro-alpine reference library version 2.0; S1 Table; deposited in the Dryad Digital Repository http://doi.org/doi:10.5061/dryad.45kf5) and a reference library based on the EMBL standard sequences release 113. The latter was created by *in silico* PCR with the program ecoPCR [45] on the EMBL standard sequences (release 113) with the *trn*L-*g* and *trn*L-*h* primers (five mismatches allowed between primer and target sequence). The filtered sequences of the diet items with their taxonomic identity as inferred by the program *ecoTag* are deposited in the Dryad Digital Repository: http://doi.org/doi:10.5061/dryad.45kf5. For all analyses we used the computing facilities provided by the Norwegian Metacenter for Computational Science (Notur).

For the development of the afro-alpine taxonomic reference library version 2.0 (S1 Table), the afro-alpine reference library version 1.0 [33] was complemented by additional taxa collected within the habitat range of the target species in the Simen Mountains (particularly from lower elevations). Vouchers of all vascular plant species were collected at the sites where groups of Walia ibex were encountered. In addition, a few small errors resulting from typos and one misidentification in the previous version of the reference library were corrected (see S1 Table). All new vouchers were identified and deposited at the National Herbarium, Addis Ababa University (AAU). Sequencing of this additional reference material was performed as described in [33]. The new library version contains sequences of the P6 loop obtained from a total of 664 specimens, representing 58 families, 172 genera and 332 species (some specimens were identified to genus only; see S1 Table for a complete taxon list). Using this library, 100% of the taxa can be identified to family, 66.7% to genus and 29.8% to species.

For the diet analysis, sequences were retained if they were present with at least 10 reads in each sample and if their minimum best identity was > 0.98 to a taxon in one of the two reference libraries, with priority given to the curated afro-alpine library (S1 Table). Automated taxonomic annotations were refined based on knowledge of the particular subset of taxa that are known to occur in the Simen Mountains, using the Flora of Ethiopia and Eritrea and the Flora of the Simen Mountains [46–51]. Sequences that were identified to the same taxon were combined into a single molecular operational taxonomic unit (MOTU), and total sequence numbers per taxon in each faecal sample were determined. Also, the forage category was established for each MOTU, differentiating between shrubs/trees, forbs and graminoids.

### Data analysis

Rarefaction analyses for DNA sequence data of the diet items were performed with the package PAST [52] in order to assess the completeness of the dietary information for each of the two target species (based on all samples of Walia ibex and domestic goats, respectively), and to evaluate the sufficiency of the sequencing depth for each individual sample. Interspecific diet variation was examined by ordination of the MOTUs per sample for each of the target species using detrended correspondence analysis (DCA [53]) in Canoco ver. 4.5 [54]. We performed the DCA using a conservative approach based purely on occurrence (presence/absence) of the MOTUs as well as a less conservative approach based on the number of sequences per MOTU. MOTUs found in only one individual were removed as these do not contribute to the comparison, but can influence outcome (rare species effect [55, 56]). We used the “down-weighting of rare species” option in Canoco. To test for the stability of the DCA ordination, we ran several analyses on the matrix, removing emerging outliers in both samples and MOTUs. To test if there are significant differences between the DCA scores of goat and Walia ibex, we scaled the scores along the first axis by their standard deviation and performed a one-way Analysis of Variance (ANOVA) with respect to species.

We further analyzed diet overlap using the simplified Morisita (or Morisita-Horn) index [57]. The calculations were performed 1) for the complete data set, 2) for the 10 most preferred diet items for each of the target species (15 MOTUs altogether), and 3) for the four most preferred MOTUs shared between Walia ibex and domestic goat. Because the vegetation change with respect to altitude is considerable and no goat samples were taken from heights above 4000 m, we performed additional calculations considering only the Walia ibex samples collected from heights comparable to those of the goat samples (16 samples).

## Results

We identified Walia ibex DNA in 23 faecal samples and domestic goat DNA in 16 samples. Sheep and klipspringer DNA was identified in six and one sample, respectively. One sample was contaminated with human DNA, and the last sample failed to amplify. Sequencing resulted in 321 745 sequence reads after filtering, of which 43.6% (140 437) came from Walia ibex samples and 56.4% (181 308) from goat samples. In total, 54 unique plant MOTUs were identified and assigned to species (22), genus (25), or higher taxonomic levels (31) (S2 Table). The faecal samples of Walia ibex and domestic goat yielded 45 and 44 plant MOTUs, respectively. The diet of Walia ibex consisted of forbs (35.9%), trees and shrubs (63.7%), and graminoids (grasses or grass-like plants; 0.4%). These proportions were similar in the goat; 42.5%, 57.0% and 0.5%, respectively.

**Fig 2:**
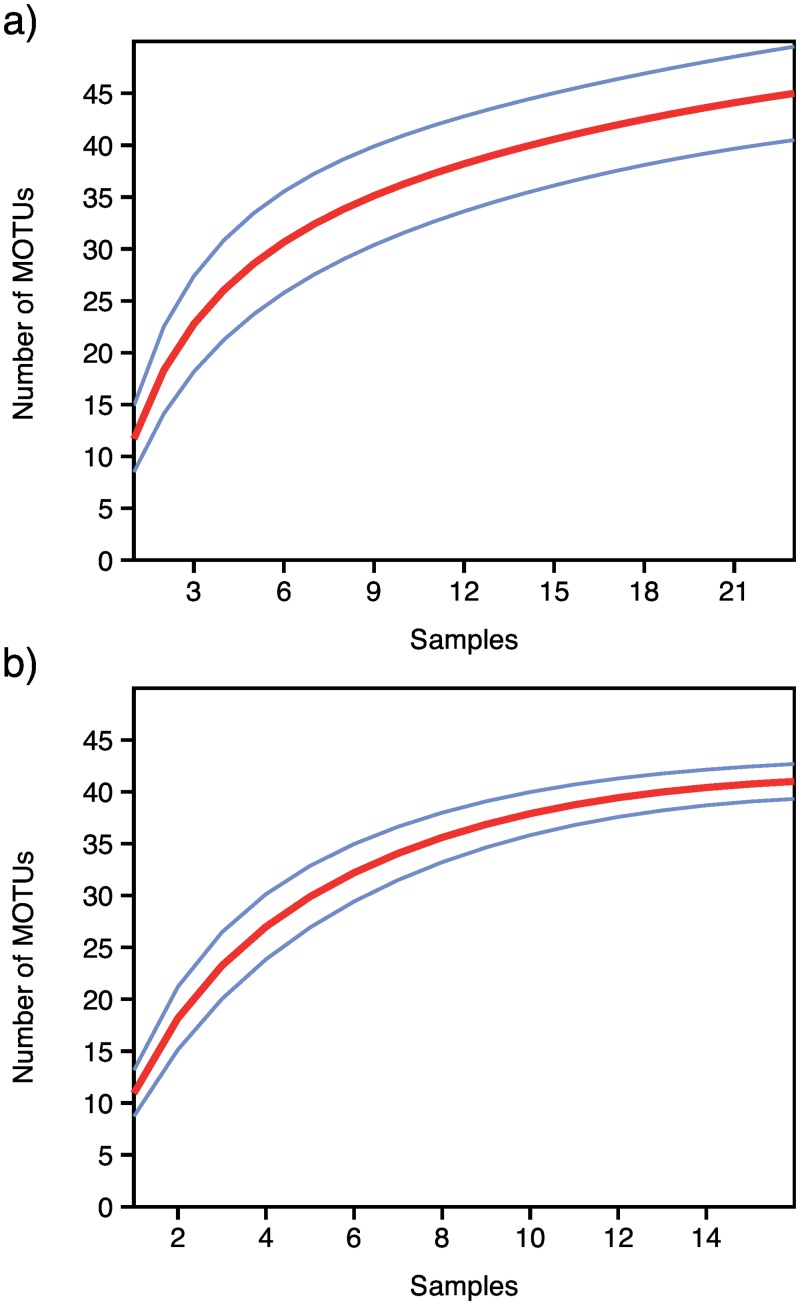
Sample-based rarefaction curves for the two species studied. a) Walia ibex diet (N = 23) and b) domestic goat diet (N = 16), for MOTU’s represented by >10 sequence reads. Blue curves represent the upper and lower limits of the 95% confidence intervals.

Within each faecal sample the number of MOTUs ranged from 4 to 28. Rarefaction curves for each individual sample reached saturation in all cases (S1 Fig), strongly indicating that the sequencing depth was sufficient to retrieve the full information from the PCR products obtained from the individual DNA extracts. The rarefaction curves for the MOTUs retrieved from each of the two target species showed that the number of MOTUs did not reach saturation for the Walia ibex (Fig 2a), which may imply that the richness of the diet of Walia ibex could increase if more samples were added to the analysis. In contrast, although the sample size was smaller for the domestic goat, the rarefaction curve appeared close to saturation (Fig 2b).

**Fig 3:**
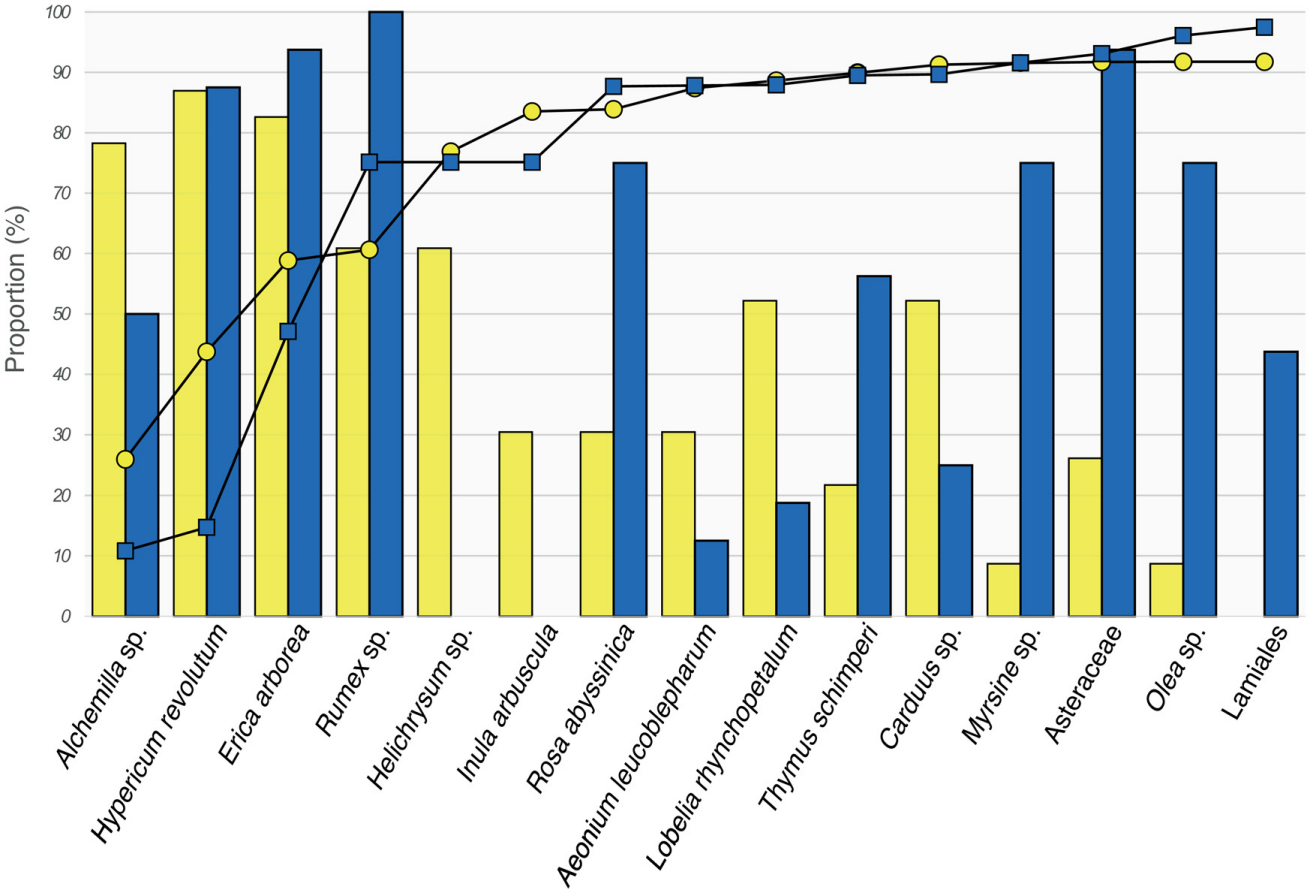
The ten most preferred diet items for the Walia ibex and domestic goat representing 15 MOTUs altogether. Yellow bars and circles for Walia ibex and blue bars and squares for domestic goat. Bars represent the proportion of faecal samples for each of the target species, in which the specified diet items were detected. Symbols connected by lines represent the cumulative percentage of diet items given as the proportion of all sequence reads from each of the target species.

The ten most preferred diet items for each of the target species, calculated from the number of samples where the item was identified and the total number of sequences retrieved for each item, represented a total of 15 MOTUs (Table 1, Fig 3). Almost 130 000 sequences from the Walia ibex samples were assigned to these 15 MOTUs, constituting 91.7% of all reads retrieved from the 23 samples. The corresponding figure for the domestic goat was close to 175 000 sequences, constituting 96.8% of all reads retrieved from the 16 samples. Five MOTUs were present in 60% or more of the Walia ibex samples: *Hypericum revolutum*, *Alchemilla* sp., *Erica arborea*, *Helichrysum* sp., and *Rumex* sp. (Table 1, Fig 3). These MOTUs represented 76.9% of all reads from the Walia ibex samples. Four of them—all but *Helichrysum* sp.—were also present at high frequencies in the domestic goat samples, constituting 75.1% of all reads from goat faeces (Table 1, Fig 3).

**Table 1:** Preferred diet items of the Walia ibex and domestic goat as interpreted from the proportional occurrence of each MOTU and its presence in individual faecal samples. Proportion is given as the percentage of sequence reads retrieved from each target species. These 15 MOTUs include the 10 most common MOTUs retrieved from each of the study species.

MOTU	# sequence reads in Walia ibex samples	# sequence reads in domestic goat samples	Proportion (%) Walia ibex	Proportion (%) domestic goat	Presence in Walia ibex samples (n = 23)	Presence in domestic goat samples (n = 16)
*Alchemilla* sp.	36438	19645	25.9	10.8	18	8
*Hypericum revolutum*	24967	7066	17.7	3.9	20	14
*Erica arborea*	21237	58679	15.1	32.3	19	15
*Helichrysum* sp.	22796	-	16.2	-	14	0
*Rumex* sp.	2516	50860	1.8	28.1	14	16
*Inula arbuscula*	9356	-	6.7	-	7	0
*Aeonium leucoblepharum*	4948	248	3.5	0.0	7	2
*Lobelia rhynchopetalum*	1734	172	1.2	0.0	12	3
*Carduus* sp.	1882	333	1.3	0.2	12	4
*Thymus schimperi*	1811	2857	1.3	1.6	5	9
*Rosa abyssinica*	504	22742	0.4	12.5	7	12
*Myrsine* sp.	382	3430	0.3	1.7	2	12
Asteraceae	214	3936	0.2	2.0	5	16
*Olea* sp.	42	5455	0.0	2.8	2	12
Lamiales	-	2509	-	1.3	0	7

Thirty-five MOTUs were shared between the two species, leaving 19 that were private to either species (S2 Table). The shared MOTUs were found in 74.2% of the Walia ibex samples and 98.2% of the goat samples. Two MOTUs private to the Walia ibex were represented by a relatively high proportion of the sequence reads (*Helichrysum* sp. and *Inula arbuscula*; 16.2% and 6.7%, respectively; S2 Table). In contrast, all MOTUs private to the domestic goat were present in low proportions (0.01–1.4%), of which Lamiales was most abundant (Fig 3, S2 Table). One MOTU (*Rosa abyssinica*) was relatively abundant in the goat samples (12.5% of sequence reads), while it was rare in the Walia ibex samples (0.4%).

**Fig 4:**
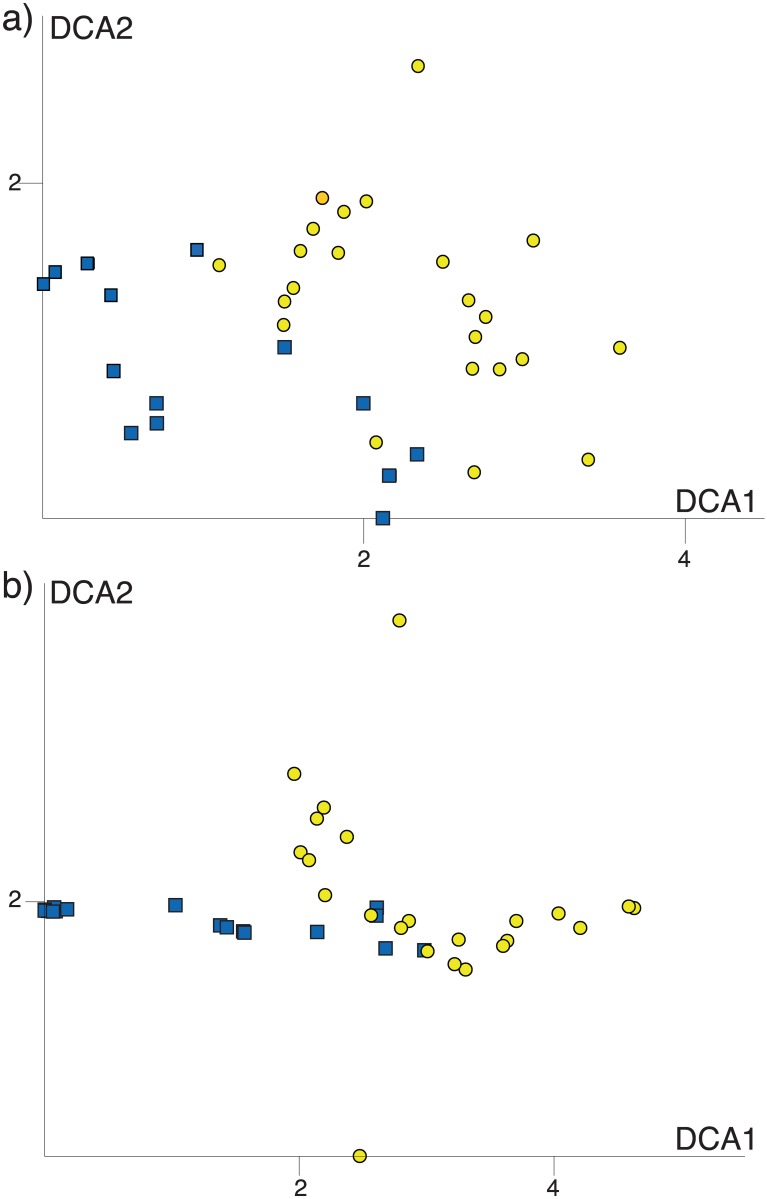
Detrended correspondence analysis (DCA) scores of diet content in samples found in Walia ibex (yellow circles) and domestic goat (blue squares). a) based on occurrence (presence/absence) and b) based on abundance of the 54 MOTUs. The DCA diagram is scaled in standard deviations units, which means that samples that are four units apart on average have a complete turnover in species (MOTU) composition. The same plant species are therefore unlikely to be found in samples that are more than four DCA units apart in the diagram.

Both DCAs resulted in a stable plot along axis 1 in all analyses (also when removing outliers), while the pattern along axis 2 was sensitive to rare MOTUs and also to rare species compositions in some Walia ibex samples (not shown). We interpret the stable DCA1 as the main gradient in diet composition, and base our interpretation of diet overlap on this axis. While there is a significant difference in DCA1 scores between goat and Walia ibex (F = 32,5; p<0.001), the visualization by standard deviation units shows that almost all sample scores were less than two standard deviation units apart from the closest individual of the other species. This corresponds to a diet overlap of 50% (Fig 4), but most individuals show a much higher degree of overlap in their diet.

**Fig 5:**
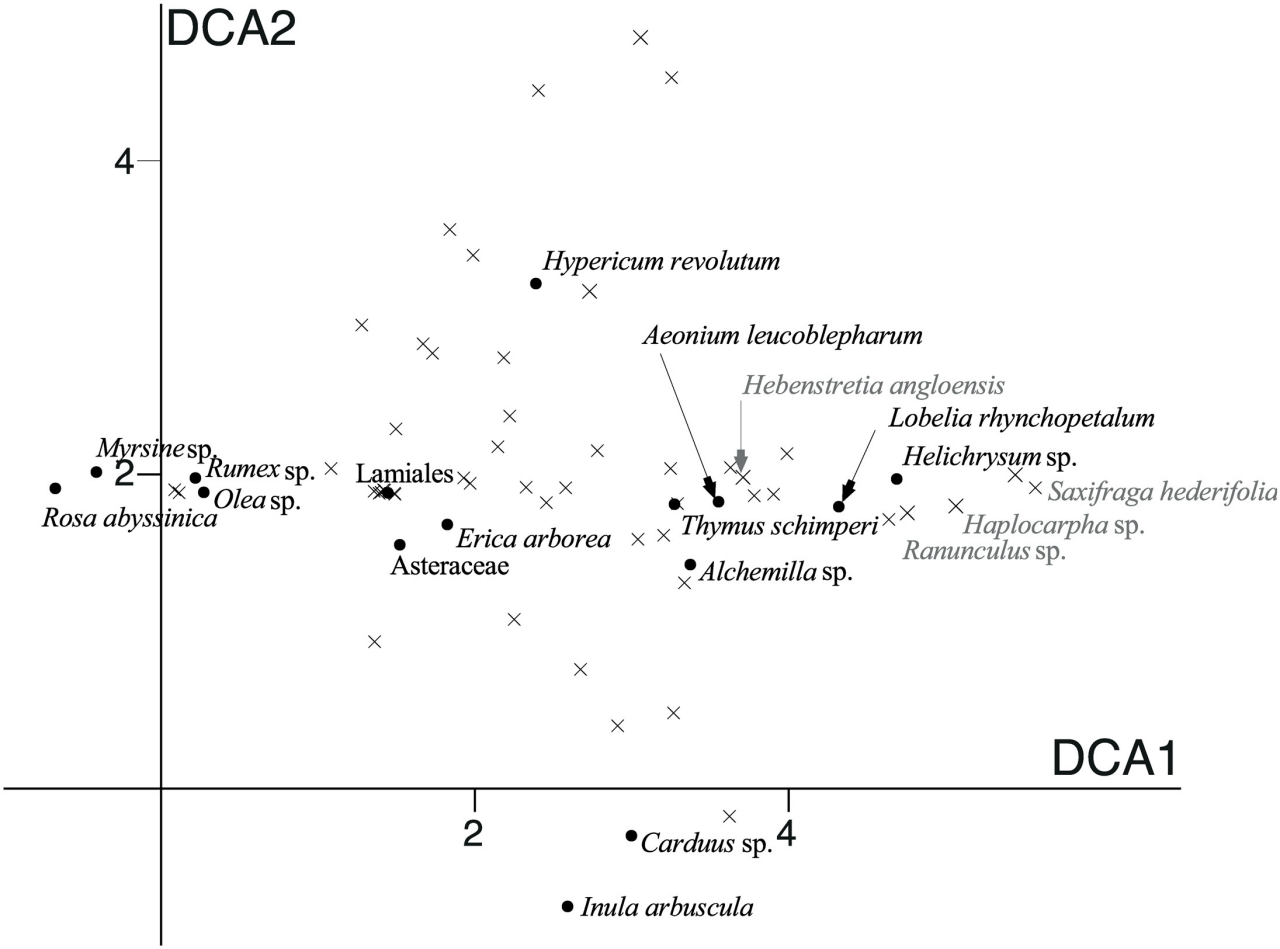
Detrended correspondence analysis (DCA) scores of the 54 plant MOTUs detected by DNA metabarcoding. The DCA diagram is scaled with standard deviations, which means that MOTU composition on average changes completely over four units along the DCA axis. MOTUs that are more than four DCA units apart in the diagram are therefore unlikely to be present in the same sample. Black dots represent the most preferred diet items (cf Table 1); grey crosses represent the position of the other MOTUs.

Plant species scores along the first two axes of the DCA using sequence count data (Fig 5) revealed that an important part of the variation was dependent on rare private diet items, despite down-weighting of rare species in the ordination procedure. This was for example the case for representatives of the genera *Saxifraga*, *Haplocarpha*, *Ranunculus*, and *Hebenstretia* that were all private to the Walia ibex, but present at very low frequencies (Fig 5, S2 Table).

**Fig 6:**
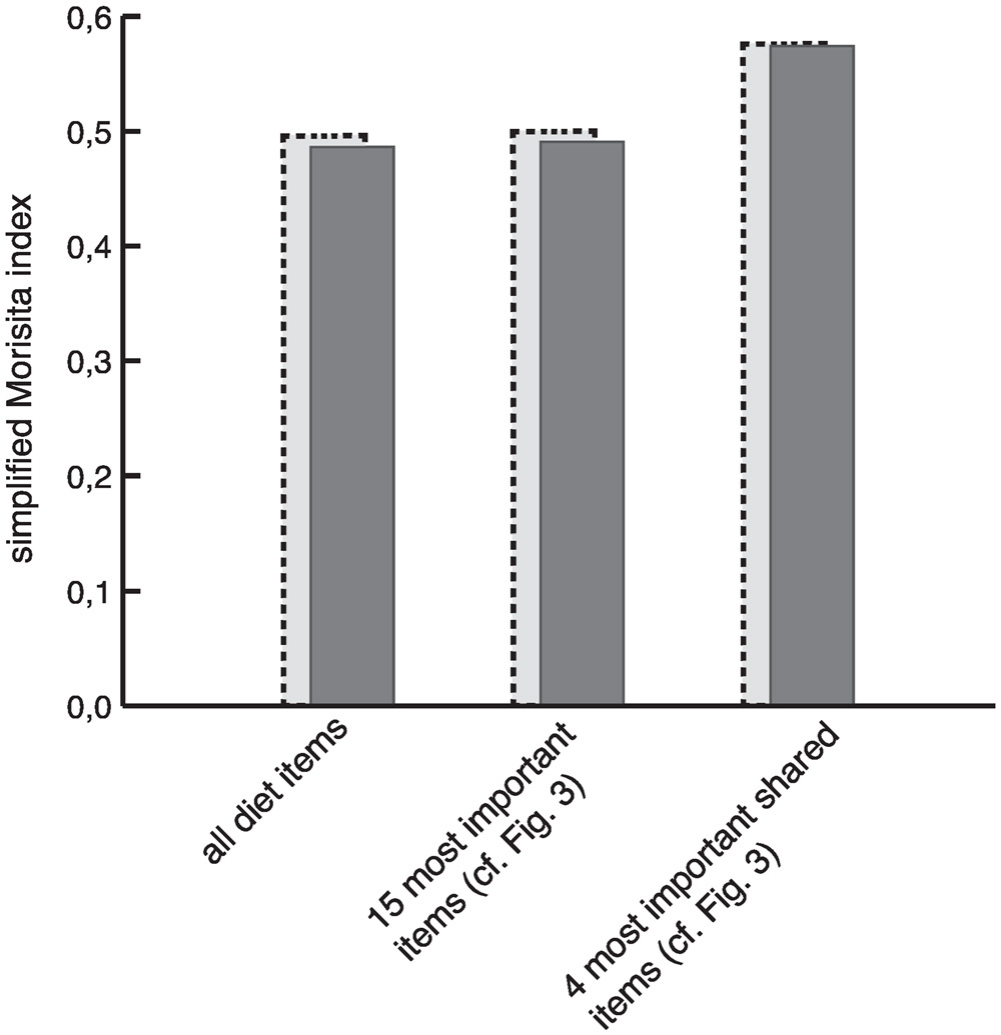
Simplified Morisita index calculated on different sets of diet items. Dark grey bars depict values calculated using all Walia ibex samples. Light grey bars with dashed frames depict values calculated using only Walia ibex samples collected at altitudes below 4000 m.

The diet overlap using the simplified Morisita index was calculated as 0.486 when considering the complete dataset (Fig 6). Restricting the analysis to the 10 most preferred diet items for each of the target species led to a negligible increase to 0.490. By further reduction of the dataset to only the four most preferred shared diet items *Alchemilla* sp., *Hypericum revolutum*, *Erica arborea* and *Rumex* sp., the Morisita index increased to 0.574. When excluding the Walia ibex samples collected above 4000 m, where no goat samples were present, the Morisita index ranged between 0.496 and 0.576 (Fig 6).

## Discussion

In this study, we analyzed the diet of the endangered Walia ibex endemic to the Simen Mountains in Ethiopia and its degree of overlap with that of domestic goat by using DNA metabarcoding of faecal samples, targeting vascular plants. The analyses revealed that the Walia ibex feeds largely on forbs and shrubs/trees, of which some, such as *Lobelia rhynchopetalum*, are endemic to Ethiopia. Graminoids were also included in the diet, but at much lower proportions. We found that although the diet of the Walia ibex is composed of a relatively large number of plant taxa, the majority of sequences retrieved stem from just a few taxa, suggesting clear dietary preferences. Therefore, although the rarefaction analysis suggests that the total number of utilized plant taxa may be even higher and could be detected by an increased sample size, we anticipate that all major diet items were identified, and that possible additional taxa are of less importance.

The metabarcoding approach identified a high number of MOTUs compared to earlier studies based on direct observations. For example, only 28 species were counted in a diet study using observational methods over a period of two years [17], compared to the 45 MOTUs identified in the diet of the Walia ibex in our samples collected during a single dry season. An observational study, in which a large part of the observations were carried out during the wet season [14] reported a higher number of Walia ibex diet items (62), but during the wet season plant species diversity is expected to be larger. In effect, the metabarcoding approach proved to be efficient in obtaining dietary information with minimal disturbance for our rare study species, notwithstanding the fact that the approach is prone to multiple biases introduced through DNA degradation and experimental procedures [58]. Such biases include potential differences in DNA preservation and in sequence lengths of the amplicon, as species with a shorter amplicon length might be preferentially amplified from degraded DNA, and shorter amplicons are in general preferentially sequenced. Biases can also be introduced in the other processing steps, e.g. through sequence variation in the primer binding sites [59]. Therefore, it is yet uncertain how closely the sequence data reflects the ingested diet in a quantitative manner. Studies comparing metabarcoding data to either known ingested diet [60] or to other diet proxies [29, 61] have found good correlations, but to account for the uncertainty, we performed the analyses using the data both quantitatively and limited to presence-absence only.

The metabarcoding data show that a limited number of taxa appear important to the Walia ibex. This finding is in accordance with an earlier observational study [14]. The most preferred taxa identified in that study contained six of the ten most preferred diet items for the Walia ibex as determined from the metabarcoding approach (Table 1), including all five diet items with high occurrence (>60%) among the Walia ibex samples (*Erica arborea*, *Helichrysum* sp., *Alchemilla* sp., *Hypericum revolutum*, *Rumex* sp.) and also *Lobelia rhynchopetalum*. The three first taxa in that list, together with *Lobelia rhynchopetalum*, appeared as important diet items in the earlier study, whereas *Hypericum revolutum*, and *Rumex* sp. were identified on only a few occasions. In effect, both the observational study and our metabarcoding study point to clear dietary preferences of the Walia ibex, and there is good concordance regarding the most important diet items.

Our results indicate a moderate diet overlap between the Walia ibex and domestic goat in the Simen Mountains National Park, which was highest when only the most abundant MOTUs were considered (Table 1, Fig 3). However, the overlap revealed in the DCA (Fig 4) appeared lower than expected, and the ANOVA on the axis scores indicated significant differences in dietary composition between the two species. This may have been caused by rare diet items, which contributed detectably to the separation of goat and Walia ibex samples in the DCA (Fig 5, S2 Table).

Species compete when they overlap in their use of limited spatial and trophic resources [62], resulting in a negative outcome for the competitively inferior species [63]. When species coexist through evolutionary time, mechanisms for resource partitioning can evolve to minimize competition and, thus, to enable coexistence [64]. However, when one of the potentially competing species is exotic to an area, there is no shared evolutionary history, and greater niche overlap can be expected [65, 66]. As such, livestock can outcompete wildlife if they co-occur in the same habitats and have similar diets [4, 67]. Empirical studies have also suggested exploitation competition as the central mechanism of interaction in wildlife-livestock systems [68, 69]. In our study, diet overlap ranged from 0.49 to 0.58, as estimated from a simplified Morisita index calculated for different sets of diet items (Fig 6). These estimates are at the high end of the range previously classified as indicating moderate overlap (0.30 to 0.59 [70]), but still below the threshold usually considered significant among sympatric species (>0.60 [68, 69]).

### Implications for conservation

Human population pressure and competition for natural resources have been increasing in and around the Simen Mountains National Park, especially over the last two decades, compromising both the livelihoods of local smallholders and the diverse fauna and flora of the Ethiopian afro-alpine ecosystem [11]. Given an increasing human population and consequently more domestic livestock in the National Park, competition between native herbivores and domestic livestock is expected to increase and native herbivores such as the Walia ibex may be forced to forage on less preferred diet items.

In our study, we were not able to include data on the abundance and density of the study species or the availability of the different plant species within the study area. Hence, it is difficult to evaluate any consequences of the moderate diet overlap on competitive interactions between the Walia ibex and domestic goat. Nevertheless, given the documented overlap in dietary preferences and the potentially devastating effect of domestic goat on plant diversity [3, 5, 6, 12], the Walia ibex may be highly vulnerable to further increases in goat numbers in the Simen Mountains National Park. This could be enhanced by an additional shift in animal husbandry from cattle to goats, which has been predicted in Africa [71]. Indeed, future conservation and management plans should consider the potential negative effects of domestic goats in the single protected area, where the endangered Walia ibex survives today.

## Supporting Information

S1 Fig Individual-based rarefaction curves of diet item sequences retrieved from a) Walia ibex samples and b) domestic goat. (TIF)

S1 Table Taxa included in the afro-alpine taxonomic *trn*L P6 loop reference library version 2.0. Specimen O-DP-30529 ET-0333-1 14.1 in reference library 1.0 [33] was removed as it had been misidentified. Nomenclature follows Flora of Ethiopia and Eritrea and Flora of Tropical East Africa and the APG III [72].
(DOCX)

S2 Table Diet molecular operational taxonomic units (MOTUs) detected in the samples. Listed MOTUs were detected more than 10 times in at least one of the two herbivore species. Occurrence refers to the number of sequence reads from the faecal samples of each species. Frequency refers to the number of samples where the MOTUs were detected.
(DOCX)
