# Supplementary figures and images for: DNA Metabarcoding Reveals Diet Overlap between the Endangered Walia Ibex and Domestic Goats - Implications for Conservation
Source: PLoS One. 2016 Jul 14;11(7):e0159133. doi: 10.1371/journal.pone.0159133 (PMC4945080; doi:10.1371/journal.pone.0159133)

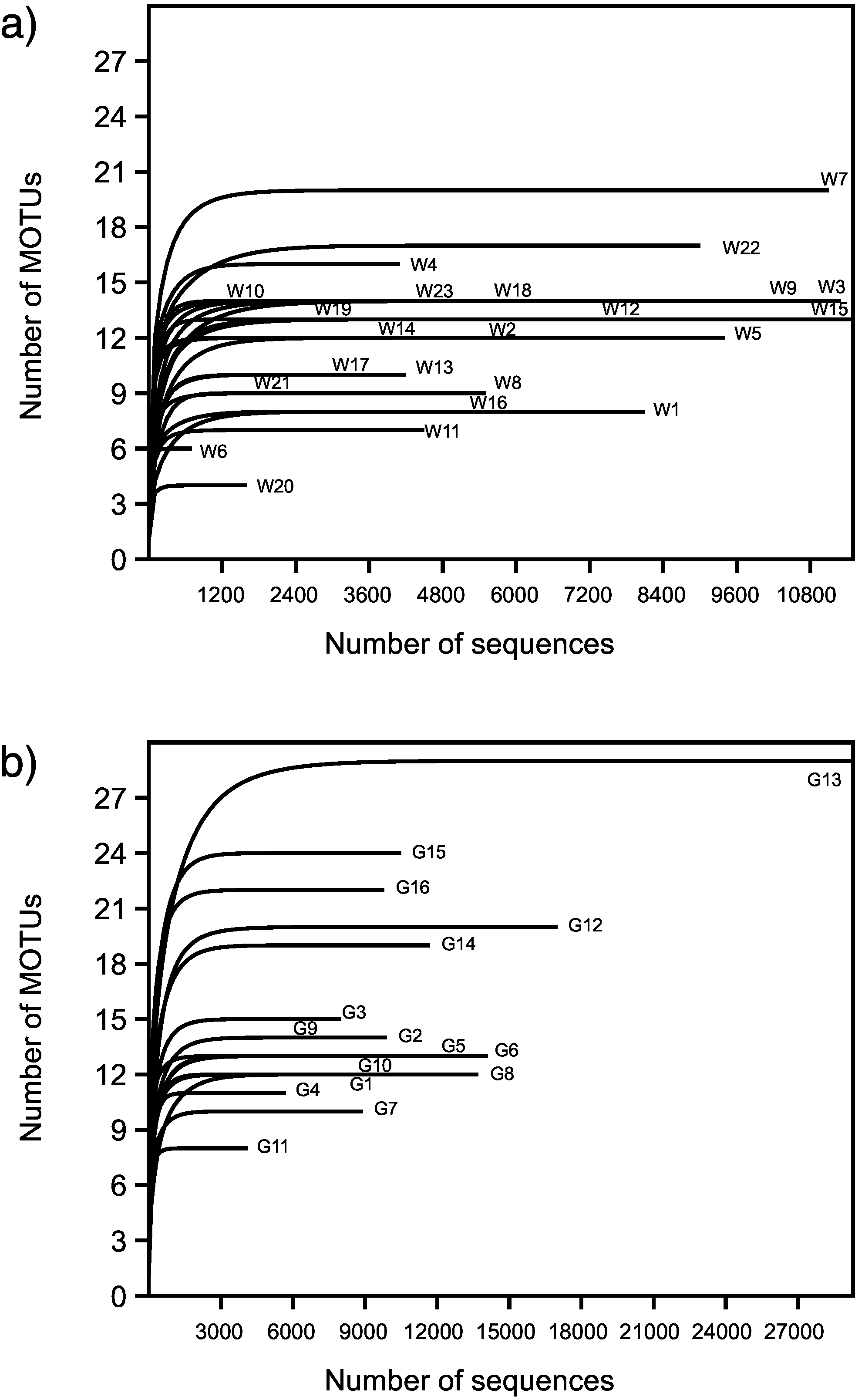

Supplement: S1 Fig — (TIF) [file pone.0159133.s001.tif]
