# Supplementary material for: DNA Metabarcoding Reveals Diet Overlap between the Endangered Walia Ibex and Domestic Goats - Implications for Conservation
Source: PLoS One. 2016 Jul 14;11(7):e0159133. doi: 10.1371/journal.pone.0159133 (PMC4945080; doi:10.1371/journal.pone.0159133)
Supplement: S1 Table — Specimen O-DP-30529 ET-0333-1 14.1 in reference library 1.0 [33] was removed as it had been misidentified. Nomenclature follows Flora of Ethiopia and Eritrea and Flora of Tropical East Africa and the APG III [72]. (DOCX) [file pone.0159133.s002.docx]

**S1 Table.** **Taxa included in the afro-alpine taxonomic *trn*L P6 loop reference library version 2.0**.  Specimen O-DP-30529 ET-0333-1 14.1 in reference library 1.0 [1] was removed as it had been misidentified. Nomenclature follows Flora of Ethiopia and Eritrea and Flora of Tropical East Africa and the APG III [2]

| \| Family \| Genus \| Species (incl. subspecies) \| No. of specimens \| \| --- \| --- \| --- \| --- \| \| Acanthaceae \| *Barleria* \| *Barleria ventricosa* \| 2 \| \|  \| *Hypoestes* \| *Hypoestes forskaolii* \| 2 \| \| Apiaceae \| *Agrocharis* \| *Agrocharis melanantha* \| 2 \| \|  \| *Heracleum* \| *Heracleum abyssincum* \| 2 \| \|  \|  \| *Heracleum elgonense* \| 2 \| \|  \| *Peucedanum* \| *Peucedanum kerstenii* \| 2 \| \|  \|  \| *Peucedanum runssoricum* \| 2 \| \|  \|  \| *Peucedanum* sp. \| 2 \| \|  \| *Pimpinella* \| *Pimpinella oreophila* \| 3 \| \|  \|  \| *Pimpinella pimpinelloides* \| 1 \| \|  \| *Sanicula* \| *Sanicula elata* \| 2 \| \|  \| *Haplosciadium* \| *Haplosciadium abyssinicum* \| 2 \| \| Apocynaceae \| *Gomphocarpus* \| *Gomphocarpus fruticosus* \| 1 \| \| Araliaceae \| *Hydrocotyle* \| *Hydrocotyle sibthorpioides* \| 1 \| \| Asparagaceae \| *Asparagus* \| *Asparagus africanus* \| 1 \| \| Asteraceae \| *Ageratum* \| *Ageratum conyzoides* \| 1 \| \|  \| *Anthemis* \| *Anthemis tigrensis* \| 2 \| \|  \| *Artemisia* \| *Artemisia abyssinica* \| 1 \| \|  \|  \| *Artemisia afra* \| 1 \| \|  \|  \| *Artemisia schimperi* \| 1 \| \|  \| *Bidens* \| *Bidens macroptera* \| 1 \| \|  \| *Carduus* \| *Carduus chamaecephalus* \| 2 \| \|  \|  \| *Carduus keniensis* \| 2 \| \|  \|  \| *Carduus leptacanthus* \| 4 \| \|  \|  \| *Carduus macracanthus* \| 2 \| \|  \|  \| *Carduus ruwenzoriensis* \| 2 \| \|  \|  \| *Carduus schimperi* \| 2 \| \|  \|  \| *Carduus schimperi* subsp. *nanus* \| 2 \| \|  \|  \| *Carduus schimperi* subsp. *platyphyllus* \| 2 \| \|  \|  \| *Carduus* sp. \| 2 \| \|  \| *Cineraria* \| *Cineraria abyssinica* \| 2 \| \|  \|  \| *Cineraria deltoidea* \| 2 \| \|  \| *Conyza* \| *Conyza hochstetterii* \| 1 \| \|  \|  \| *Conyza schimperi* \| 1 \| \|  \|  \| *Conyza subscaposa* \| 1 \| \|  \|  \| *Conyza* sp. \| 1 \| \|  \| *Cotula* \| *Cotula abyssinica* \| 1 \| \|  \|  \| *Cotula cryptocephala* \| 2 \| \|  \| *Crepis* \| *Crepis dianthoseris* \| 1 \| \|  \|  \| *Crepis foetida* \| 1 \| \|  \|  \| *Crepis rueppellii* \| 1 \| \|  \|  \| *Crepis* sp. \| 2 \| \|  \| *Dendrosenecio* \| *Dendrosenecio adnivalis* subsp. *adnivalis* \| 2 \| \|  \|  \| *Dendrosenecio battiscombei* \| 2 \| \|  \|  \| *Dendrosenecio brassiciformis* \| 2 \| \|  \|  \| *Dendrosenecio cheranganensis* subsp. *cheranganensis* \| 2 \| \|  \|  \| *Dendrosenecio cheranganensis* subsp. *dalei* \| 2 \| \|  \|  \| *Dendrosenecio elgonensis* subsp. *barbatipes* \| 2 \| \|  \|  \| *Dendrosenecio elgonensis* subsp. *elgonensis* \| 2 \| \|  \|  \| *Dendrosenecio erici-rosenii* subsp. *alticola* \| 2 \| \|  \|  \| *Dendrosenecio erici-rosenii* subsp. *erici-rosenii* \| 2 \| \|  \|  \| *Dendrosenecio keniensis* \| 2 \| \|  \|  \| *Dendrosenecio keniensisxkeniodendron* \| 2 \| \|  \|  \| *Dendrosenecio keniodendron* \| 4 \| \|  \|  \| *Dendrosenecio kilimanjari* subsp. *cottonii* \| 2 \| \|  \|  \| *Dendrosenecio kilimanjari* subsp. *kilimanjari* \| 2 \| \|  \|  \| *Dendrosenecio meruensis* \| 2 \| \|  \|  \| *Dichrocephala chrysanthemifolia* \| 2 \| \|  \| *Echinops* \| *Echinops buhaitensis* \| 2 \| \|  \|  \| *Echinops macrochaetus* \| 1 \| \|  \| *Erigeron* \| *Erigeron alpinus* \| 2 \| \|  \| *Euryops* \| *Euryops brownei* \| 2 \| \|  \|  \| *Euryops dacrydioides* \| 2 \| \|  \|  \| *Euryops elgonensis* \| 2 \| \|  \|  \| *Euryops pinifolia* \| 2 \| \|  \|  \| *Euryops prostratus* \| 2 \| \|  \| *Gnaphalium* \| *Gnaphalium unionis* \| 2 \| \|  \| *Guizotia* \| *Guizotia scabra* \| 1 \| \|  \| *Haplocarpha* \| *Haplocarpha hastata* \| 2 \| \|  \|  \| *Haplocarpha rueppellii* \| 3 \| \|  \|  \| *Haplocarpha schimperi* \| 1 \| \|  \| *Helichrysum* \| *Helichrysum brownie* \| 1 \| \|  \|  \| *Helichrysum citrispinum* \| 1 \| \|  \|  \| *Helichrysum formosissimum* \| 2 \| \|  \|  \| *Helichrysum forskahlii* \| 2 \| \|  \|  \| *Helichrysum globosum* \| 2 \| \|  \|  \| *Helichrysum gofense* \| 2 \| \|  \|  \| *Helichrysum horridum* \| 2 \| \|  \|  \| *Helichrysum kilimanjari* \| 1 \| \|  \|  \| *Helichrysum odoratissimum* \| 1 \| \|  \|  \| *Helichrysum splendidum* \| 2 \| \|  \|  \| *Helichrysum stuhlmannii* \| 2 \| \|  \|  \| *Helichrysum* sp. \| 5 \| \|  \| *Inula* \| *Inula donfertiflora* \| 1 \| \|  \| *Lactuca* \| *Lactuca inermis* \| 2 \| \|  \| *Laggera* \| *Laggera crispata* \| 1 \| \|  \| *Plectocephalus* \| *Plectocephalus varians* \| 2 \| \|  \| *Senecio* \| *Senecio balensis* \| 2 \| \|  \|  \| *Senecio farinaceus* \| 1 \| \|  \|  \| *Senecio fresenii* \| 2 \| \|  \|  \| *Senecio jacksonii* \| 2 \| \|  \|  \| *Senecio meyeri-johannis* \| 1 \| \|  \|  \| *Senecio nanus* \| 1 \| \|  \|  \| *Senecio polyadenus* \| 2 \| \|  \|  \| *Senecio purtschelleri* \| 1 \| \|  \|  \| *Senecio rhammatophyllus* \| 2 \| \|  \|  \| *Senecio sabinjoensis* \| 2 \| \|  \|  \| *Senecio schultzii* \| 2 \| \|  \|  \| *Senecio schweinfurthii* \| 2 \| \|  \|  \| *Senecio subsessilis* \| 2 \| \|  \|  \| *Senecio telekii* \| 2 \| \|  \|  \| *Senecio transmarinus* \| 2 \| \|  \|  \| *Senecio unionis* \| 2 \| \|  \|  \| *Senecio* sp. \| 3 \| \|  \| *Sonchus* \| *Sonchus melanolepis* \| 2 \| \|  \| *Sphaeranthus* \| *Sphaeranthus suaveolens* \| 1 \| \|  \| *Stoebe* \| *Stoebe kilimandscharica* \| 1 \| \|  \| *Ursinia* \| *Ursinia nana* \| 2 \| \|  \| *Vernonia* \| *Vernonia arnygdalina* \| 1 \| \|  \|  \| *Vernonia myriantha* \| 1 \| \| Bignoniaceae \| *Stereospermum* \| *Stereospermum kunthianum* \| 1 \| \| Boraginaceae \| *Cynoglossum* \| *Cynoglossum* sp. \| 1 \| \|  \| *Myosotis* \| *Myosotis keniensis* \| 2 \| \| Brassicaceae \| *Arabidopsis* \| *Arabidopsis thaliana* \| 2 \| \|  \| *Arabis* \| *Arabis alpina* \| 2 \| \|  \| *Barbarea* \| *Barbarea intermedia* \| 2 \| \|  \| *Cardamine* \| *Cardamine hirsuta* \| 1 \| \|  \|  \| *Cardamine obliqua* \| 2 \| \|  \| *Erophila* \| *Erophila verna* \| 1 \| \|  \| *Oreophyton* \| *Oreophyton falcatum* \| 1 \| \|  \| *Subularia* \| *Subularia monticola* \| 2 \| \|  \| *Thlaspi* \| *Thlaspi alliaceum* \| 2 \| \| Campanulaceae \| *Campanula* \| *Campanula edulis* \| 2 \| \|  \| *Lobelia* \| *Lobelia aberdarica* \| 3 \| \|  \|  \| *Lobelia acrochilus* \| 1 \| \|  \|  \| *Lobelia bambuseti* \| 2 \| \|  \|  \| *Lobelia bequaertii* \| 2 \| \|  \|  \| *Lobelia burtii* subsp. *meruensis* \| 2 \| \|  \|  \| *Lobelia deckenii* \| 2 \| \|  \|  \| *Lobelia deckenii* subsp. *burtii* \| 1 \| \|  \|  \| *Lobelia deckenii* subsp. *deckenii* \| 1 \| \|  \|  \| *Lobelia deckenii* subsp. *elgonensis* \| 1 \| \|  \|  \| *Lobelia erlangeriana* \| 1 \| \|  \|  \| *Lobelia gibberoa* \| 3 \| \|  \|  \| *Lobelia gregoriana* subsp. *elgonensis* \| 4 \| \|  \|  \| *Lobelia gregoriana* subsp. *sattima* \| 1 \| \|  \|  \| *Lobelia lindblomii* \| 2 \| \|  \|  \| *Lobelia mildbraedii* \| 2 \| \|  \|  \| *Lobelia rhynchopetalum* \| 3 \| \|  \|  \| *Lobelia schimperi* \| 1 \| \|  \|  \| *Lobelia stuhlmannii* \| 2 \| \|  \|  \| *Lobelia telekii* \| 2 \| \|  \|  \| *Lobelia thuliniana* \| 2 \| \|  \|  \| *Lobelia welwitschii* \| 2 \| \|  \|  \| *Lobelia wollastonii* \| 2 \| \|  \| *Monopsis* \| *Monopsis stellarioides* \| 1 \| \|  \| *Wahlenbergia* \| *Wahlenbergia krebsii* subsp. *arguta* \| 1 \| \|  \|  \| *Wahlenbergia silenoides* \| 1 \| \| Capparaceae \| *Capparis* \| *Capparis tomentosa* \| 1 \| \| Caprifoliaceae \| *Dipsacus* \| *Dipsacus pinnatifidus* \| 2 \| \|  \| *Scabiosa* \| *Scabiosa columbaria* \| 4 \| \|  \| *Valeriana* \| *Valeriana kilimandscharica* \| 2 \| \|  \| *Valerianella* \| *Valerianella microcarpa* \| 1 \| \| Caryophyllaceae \| *Cerastium* \| *Cerastium indicum* \| 1 \| \|  \|  \| *Cerastium octandrum* \| 2 \| \|  \| *Dianthus* \| *Dianthus longiglumis* \| 3 \| \|  \| *Herniaria* \| *Herniaria abyssinica* \| 1 \| \|  \| *Lychnis* \| *Lychnis abyssinica* \| 1 \| \|  \|  \| *Lychnis rotundifolia* \| 1 \| \|  \| *Minuartia* \| *Minuartia filifolia* \| 1 \| \|  \| *Paronychia* \| *Paronychia bryoides* \| 2 \| \|  \| *Sagina* \| *Sagina abyssinica* \| 2 \| \|  \|  \| *Sagina afroalpina* \| 2 \| \|  \| *Silene* \| *Silene abyssinica* \| 1 \| \|  \| *Silene* \| *Silene burchellii* \| 2 \| \|  \|  \| *Silene flammulifolia* \| 1 \| \|  \|  \| *Silene macrosolen* \| 2 \| \|  \|  \| *Silene* sp. \| 1 \| \|  \| *Stellaria* \| *Stellaria media* \| 2 \| \|  \|  \| *Stellaria sennii* \| 2 \| \| Celastraceae \| *Maytenus* \| *Maytenus gracilipes* \| 1 \| \| Colchicaceae \| *Merendera* \| *Merendera schimperiana* \| 2 \| \| Crassulaceae \| *Aeonium* \| *Aeonium leucoblepharum* \| 1 \| \|  \| *Crassula* \| *Crassula granvikii* \| 1 \| \|  \|  \| *Crassula schimperi* \| 2 \| \|  \| *Sedum* \| *Sedum crassularia* \| 2 \| \|  \|  \| *Sedum meyeri-johannis* \| 1 \| \|  \|  \| *Sedum mooneyi* \| 2 \| \|  \| *Umbilicus* \| *Umbilicus botryoides* \| 2 \| \| Cyperaceae \| *Bulbostylis* \| *Bulbostylis atrosanguinez* \| 1 \| \|  \| *Carex* \| *Carex fischeri* \| 1 \| \|  \|  \| *Carex monostachya* \| 2 \| \|  \|  \| *Carex runssoroensis* \| 2 \| \|  \|  \| *Carex simensis* \| 3 \| \|  \|  \| *Carex* sp. \| 1 \| \|  \| *Cyperus* \| *Cyperus elegantulus* \| 1 \| \|  \|  \| *Cyperus plateilema* \| 2 \| \|  \| *Ficinia* \| *Ficinia* sp. \| 1 \| \|  \|  \| *Ficinia stolonifera* \| 2 \| \|  \| *Isolepis* \| *Isolepis costata* \| 2 \| \|  \|  \| *Isolepis fluitans* \| 1 \| \|  \|  \| *Isolepis fluitans* subsp. *ruwenzoriensis* \| 1 \| \| Dennstaedtiaceae \| *Pteridium* \| *Pteridium aquilinum* \| 1 \| \| Dryopteridaceae \| *Dryopteris* \| *Dryopteris lewalleana* \| 1 \| \|  \|  \| *Dryopteris* sp. \| 2 \| \| Ericaceae \| *Erica* \| *Erica arborea* \| 3 \| \|  \|  \| *Erica johnstonii* \| 2 \| \|  \|  \| *Erica tenuipilosa* \| 1 \| \|  \|  \| *Erica tenuipilosa* subsp. *spicata* \| 2 \| \|  \|  \| *Erica trimera* subsp. *trimera* \| 1 \| \| Eriocaulaceae \| *Eriocaulon* \| *Eriocaulon schimperi* \| 1 \| \|  \|  \| *Eriocaulon volkensii* \| 2 \| \|  \|  \| *Eriocaulon* sp. \| 1 \| \| Euphorbiaceae \| *Euphorbia* \| *Euphorbia schimperiana* \| 1 \| \| Fabaceae \| *Argyrolobium* \| *Argyrolobium rupestre* \| 1 \| \|  \|  \| *Argyrolobium schimperianum* \| 1 \| \|  \| *Trifolium* \| *Trifolium acaule* \| 6 \| \|  \|  \| *Trifolium burchellianum* subsp. *johnstonii* \| 4 \| \|  \|  \| *Trifolium calocephalum* \| 2 \| \|  \|  \| *Trifolium campestre* \| 1 \| \|  \|  \| *Trifolium cryptopodium* \| 4 \| \|  \|  \| *Trifolium rueppellianum* \| 1 \| \|  \|  \| *Trifolium simense* \| 2 \| \|  \|  \| *Trifolium* sp. \| 1 \| \| Gentianaceae \| *Swertia* \| *Swertia abyssinica* \| 1 \| \|  \|  \| *Swertia adolfi-friderici* \| 1 \| \|  \|  \| *Swertia brownii* \| 1 \| \|  \|  \| *Swertia crassiuscula* subsp. *crassiuscula* \| 2 \| \|  \|  \| *Swertia crassiuscula* subsp. *robusta* \| 1 \| \|  \|  \| *Swertia engleri* \| 2 \| \|  \|  \| *Swertia engleri* subsp. *woodii* \| 2 \| \|  \|  \| *Swertia macrosepala* subsp. *macrosepala* \| 1 \| \|  \|  \| *Swertia macrosepala* subsp. *microsperma* \| 1 \| \|  \|  \| *Swertia pumila* \| 1 \| \|  \|  \| *Swertia quartiniana* \| 1 \| \|  \|  \| *Swertia schimperi* \| 2 \| \|  \|  \| *Swertia subnivalis* \| 1 \| \|  \|  \| *Swertia uniflora* \| 1 \| \| Geraniaceae \| *Geranium* \| *Geranium arabicum* \| 6 \| \|  \|  \| *Geranium kilimandscharicum* \| 1 \| \| Hypericaceae \| *Hypericum* \| *Hypericum quartinianum* \| 1 \| \|  \|  \| *Hypericum revolutum* \| 4 \| \|  \|  \| *Hypericum* sp. \| 3 \| \| Iridaceae \| *Dierama* \| *Dierama cupuliflorum* \| 4 \| \|  \| *Gladiolus* \| *Gladiolus watsonioides* \| 1 \| \|  \| *Hesperantha* \| *Hesperantha petitiana* \| 2 \| \|  \| *Romulea* \| *Romulea congoensis* \| 2 \| \|  \|  \| *Romulea fischeri* \| 2 \| \| Juncaceae \| *Juncus* \| *Juncus capitatus* \| 1 \| \|  \| *Luzula* \| *Luzula abyssinica* \| 4 \| \|  \|  \| *Luzula johnstonii* \| 1 \| \| Lamiaceae \| *Ajuga* \| *Ajuga integrifolia* \| 1 \| \|  \| *Leonotis* \| *Leonotis ocymifolia* \| 1 \| \|  \| *Nepeta* \| *Nepeta azurea* \| 1 \| \|  \| *Salvia* \| *Salvia nilotica* \| 2 \| \|  \| *Satureja* \| *Satureja abyssinica* \| 2 \| \|  \|  \| *Satureja kilimandschari* \| 1 \| \|  \|  \| *Satureja pseudosimensis* \| 2 \| \|  \|  \| *Satureja punctata* subsp. *punctata* \| 1 \| \|  \|  \| *Satureja simensis* \| 1 \| \|  \|  \| *Satureja uhligii* \| 1 \| \|  \|  \| *Satureja* sp. \| 2 \| \|  \| *Thymus* \| *Thymus schimperi* \| 2 \| \|  \|  \| *Thymus serrulatus* \| 2 \| \| Lycopodiaceae \| *Huperzia* \| *Huperzia saururus* \| 1 \| \| Lythraceae \| *Lythrum* \| *Lythrum rotundifolium* \| 1 \| \| Malvaceae \| *Malva* \| *Malva verticillata* \| 2 \| \| Montiaceae \| *Montia* \| *Montia fontana* \| 1 \| \| Myricaceae \| *Myrica* \| *Myrica salicifolia* \| 1 \| \| Oleaceae \| *Jasminum* \| *Jasminum abyssinicum* \| 1 \| \|  \|  \| *Jasminum grandiflorum* \| 1 \| \|  \| *Olea* \| *Olea welwitschii* \| 1 \| \| Onagraceae \| *Epilobium* \| *Epilobium hirsutum* \| 1 \| \|  \|  \| *Epilobium stereophyllum* \| 2 \| \| Orchidaceae \| *Disa* \| *Disa stairsii* \| 2 \| \|  \|  \| *Disa* sp. \| 1 \| \|  \| *Habenaria* \| *Habenaria eggelingii* \| 1 \| \| Orobanchaceae \| *Bartsia* \| *Bartsia decurva* \| 3 \| \|  \|  \| *Bartsia longiflora* \| 2 \| \|  \|  \| *Bartsia longiflora* subsp. *longiflora* \| 1 \| \|  \|  \| *Bartsia longiflora* subsp. *macrophylla* \| 2 \| \| Oxalidaceae \| *Oxalis* \| *Oxalis corniculata* \| 1 \| \|  \|  \| *Oxalis obliquifolia* \| 2 \| \|  \|  \| *Oxalis procumbens* subsp. *procumbens* \| 2 \| \|  \|  \| *Oxalis* sp. \| 2 \| \| Plantaginaceae \| *Callitriche* \| *Callitriche oreophila* \| 2 \| \|  \|  \| *Callitriche vulcanicola* \| 4 \| \|  \| *Kickxia* \| *Kickxia* \| 1 \| \|  \| *Plantago* \| *Plantago afra* \| 2 \| \|  \|  \| *Plantago lanceolata* \| 1 \| \|  \| *Sibthorpia* \| *Sibthorpia* sp. \| 1 \| \|  \| *Veronica* \| *Veronica anagallis-aquatica* \| 3 \| \|  \|  \| *Veronica arvensis* \| 2 \| \|  \|  \| *Veronica glandulosa* \| 2 \| \|  \|  \| *Veronica gunae* \| 1 \| \|  \|  \| *Veronica* sp. \| 1 \| \| Poaceae \| *Agrostis* \| *Agrostis gracilifolia* \| 1 \| \|  \|  \| *Agrostis gracilifolia* subsp. *gracilifolia* \| 1 \| \|  \|  \| *Agrostis quinqueseta* \| 1 \| \|  \|  \| *Agrostis sclerophylla* \| 1 \| \|  \|  \| *Agrostis trachyphylla* \| 1 \| \|  \|  \| *Agrostis volkensii* \| 2 \| \|  \|  \| *Agrostis* sp. \| 1 \| \|  \| *Aira* \| *Aira caryophyllea* \| 1 \| \|  \| *Alopecurus* \| *Alopecurus baptarrhenius* \| 1 \| \|  \| *Andropogon* \| *Andropogon amethystinus* \| 3 \| \|  \|  \| *Andropogon chrysostachyus* \| 1 \| \|  \|  \| *Andropogon lima* \| 2 \| \|  \| *Anthoxanthum* \| *Anthoxanthum nivale* \| 4 \| \|  \| *Avenella* \| *Avenella flexuosa* \| 2 \| \|  \| *Bromus* \| *Bromus leptoclados* \| 2 \| \|  \| *Calamagrostis* \| *Calamagrostis* sp. \| 1 \| \|  \| *Deschampsia* \| *Deschampsia cespitosa* \| 2 \| \|  \| *Festuca* \| *Festuca abyssinica* \| 6 \| \|  \|  \| *Festuca macrophylla* \| 2 \| \|  \|  \| *Festuca obturbans* \| 1 \| \|  \|  \| *Festuca pilgeri* \| 1 \| \|  \|  \| *Festuca* sp. \| 1 \| \|  \| *Helictotrichon* \| *Helictotrichon* sp. \| 2 \| \|  \| *Koeleria* \| *Koeleria capensis* \| 2 \| \|  \| *Pennisetum* \| *Pennisetum humile* \| 2 \| \|  \| *Pentaschistis* \| *Pentaschistis* sp. \| 6 \| \|  \| *Poa* \| *Poa annua* \| 1 \| \|  \|  \| *Poa schimperiana* \| 4 \| \|  \| *Polypogon* \| *Polypogon schimperianus* \| 2 \| \|  \| *Rytidosperma* \| *Rytidosperma grandiflorum* \| 2 \| \|  \|  \| *Rytidosperma subulata* \| 2 \| \|  \| *Vulpia* \| *Vulpia bromoides* \| 1 \| \| Polygalaceae \| *Polygala* \| *Polygala steudneri* \| 3 \| \| Polygonaceae \| *Persicaria* \| *Persicaria nepalensis* \| 1 \| \|  \| *Polygonum* \| *Polygonum afromontanum* \| 4 \| \|  \| *Rumex* \| *Rumex nepalensis* \| 1 \| \|  \|  \| *Rumex nervosus* \| 1 \| \| Primulaceae \| *Anagallis* \| *Anagallis serpens* subsp. *serpens* \| 3 \| \|  \| *Myrsine* \| *Myrsine melanophloeos* \| 2 \| \|  \| *Primula* \| *Primula verticillata* \| 3 \| \| Proteaceae \| *Protea* \| *Protea gaguedi* \| 1 \| \| Pteridaceae \| *Adiantum* \| *Adiantum thalictroides* \| 1 \| \| Ranunculaceae \| *Anemone* \| *Anemone thomsonii* \| 2 \| \|  \|  \| *Anemone* sp. \| 2 \| \|  \| *Delphinium* \| *Delphinium wellbyi* \| 2 \| \|  \|  \| *Delphinium* sp. \| 2 \| \|  \| *Ranunculus* \| *Ranunculus distrias* \| 1 \| \|  \|  \| *Ranunculus multifidus* \| 1 \| \|  \|  \| *Ranunculus oligocarpus* \| 2 \| \|  \|  \| *Ranunculus oreophytus* \| 4 \| \|  \|  \| *Ranunculus stagnalis* \| 2 \| \|  \|  \| *Ranunculus trichophyllus* \| 1 \| \|  \|  \| *Ranunculus volkensii* \| 2 \| \|  \|  \| *Ranunculus* sp. \| 1 \| \| Rosaceae \| *Alchemilla* \| *Alchemilla abyssinica* \| 2 \| \|  \|  \| *Alchemilla argyrophylla* subsp. *argyrophylla* \| 2 \| \|  \|  \| *Alchemilla argyrophylla* subsp. *argyrophylloides* \| 1 \| \|  \|  \| *Alchemilla ellenbeckii* subsp. *ellenbeckii* \| 2 \| \|  \|  \| *Alchemilla haumannii* \| 2 \| \|  \|  \| *Alchemilla johnstonii* \| 2 \| \|  \|  \| *Alchemilla microbetula* \| 3 \| \|  \|  \| *Alchemilla pedata* \| 2 \| \|  \|  \| *Alchemilla stuhlmannii* \| 2 \| \|  \|  \| *Alchemilla triphylla* \| 2 \| \|  \|  \| *Alchemilla* sp. \| 2 \| \|  \| *Aphanes* \| *Aphanes bachitii* \| 2 \| \|  \| *Rosa* \| *Rosa abyssinica* \| 1 \| \| Rubiaceae \| *Anthospermum* \| *Anthospermum usambarense* \| 1 \| \|  \| *Galiniera* \| *Galiniera saxifraga* \| 1 \| \|  \| *Galium* \| *Galium acrophyum* \| 3 \| \|  \|  \| *Galium ruwenzoriense* \| 2 \| \|  \|  \| *Galium simense* \| 2 \| \|  \|  \| *Galium* sp. \| 2 \| \|  \| *Phyllopentas* \| *Phyllopentas schimperiana* \| 1 \| \| Rutaceae \| *Clausena* \| *Clausena anisata* \| 1 \| \| Saxifragaceae \| *Saxifraga* \| *Saxifraga hederifolia* \| 1 \| \| Scrophulariaceae \| *Celsia* \| *Rhabdotosperma scrophulariifolia* \| 1 \| \|  \| *Hebenstretia* \| *Hebenstretia angolensis* \| 1 \| \|  \|  \| *Hebenstretia* sp. \| 2 \| \|  \| *Limosella* \| *Limosella major* \| 2 \| \|  \| *Verbascum* \| *Verbascum sedgwickianum* \| 1 \| \|  \|  \| *Verbascum sinaiticum* \| 1 \| \| Solanaceae \| *Discopodium* \| *Discopodium penninervium* \| 1 \| \|  \| *Solanum* \| *Solanum anguivi* \| 1 \| \| Stilbaceae \| *Halleria* \| *Halleria lucida* \| 1 \| \|  \| *Nuxia* \| *Nuxia congesta* \| 1 \| \| Urticaceae \| *Parietaria* \| *Parietaria debilis* \| 2 \| \| Verbenaceae \| *Verbena* \| *Verbena officinalis* \| 1 \| \| Violaceae \| *Viola* \| *Viola abyssinica* \| 2 \| \|  \| *Viola* \| *Viola eminii* \| 2 \| \| Xanthorrhoeaceae \| *Kniphofia* \| *Kniphofia foliosa* \| 2 \| \|  \|  \| *Kniphofia isoetifolia* \| 2 \| \|  \|  \| *Kniphofia thomsonii* \| 2 \| \|  \|  \| *Kniphofia* sp. \| 2 \| \|  \|  \|  \|  \| |
| --- | --- | --- | --- | --- | --- | --- | --- | --- | --- | --- | --- | --- | --- | --- | --- | --- | --- | --- | --- | --- | --- | --- | --- | --- | --- | --- | --- | --- | --- | --- | --- | --- | --- | --- | --- | --- | --- | --- | --- | --- | --- | --- | --- | --- | --- | --- | --- | --- | --- | --- | --- | --- | --- | --- | --- | --- | --- | --- | --- | --- | --- | --- | --- | --- | --- | --- | --- | --- | --- | --- | --- | --- | --- | --- | --- | --- | --- | --- | --- | --- | --- | --- | --- | --- | --- | --- | --- | --- | --- | --- | --- | --- | --- | --- | --- | --- | --- | --- | --- | --- | --- | --- | --- | --- | --- | --- | --- | --- | --- | --- | --- | --- | --- | --- | --- | --- | --- | --- | --- | --- | --- | --- | --- | --- | --- | --- | --- | --- | --- | --- | --- | --- | --- | --- | --- | --- | --- | --- | --- | --- | --- | --- | --- | --- | --- | --- | --- | --- | --- | --- | --- | --- | --- | --- | --- | --- | --- | --- | --- | --- | --- | --- | --- | --- | --- | --- | --- | --- | --- | --- | --- | --- | --- | --- | --- | --- | --- | --- | --- | --- | --- | --- | --- | --- | --- | --- | --- | --- | --- | --- | --- | --- | --- | --- | --- | --- | --- | --- | --- | --- | --- | --- | --- | --- | --- | --- | --- | --- | --- | --- | --- | --- | --- | --- | --- | --- | --- | --- | --- | --- | --- | --- | --- | --- | --- | --- | --- | --- | --- | --- | --- | --- | --- | --- | --- | --- | --- | --- | --- | --- | --- | --- | --- | --- | --- | --- | --- | --- | --- | --- | --- | --- | --- | --- | --- | --- | --- | --- | --- | --- | --- | --- | --- | --- | --- | --- | --- | --- | --- | --- | --- | --- | --- | --- | --- | --- | --- | --- | --- | --- | --- | --- | --- | --- | --- | --- | --- | --- | --- | --- | --- | --- | --- | --- | --- | --- | --- | --- | --- | --- | --- | --- | --- | --- | --- | --- | --- | --- | --- | --- | --- | --- | --- | --- | --- | --- | --- | --- | --- | --- | --- | --- | --- | --- | --- | --- | --- | --- | --- | --- | --- | --- | --- | --- | --- | --- | --- | --- | --- | --- | --- | --- | --- | --- | --- | --- | --- | --- | --- | --- | --- | --- | --- | --- | --- | --- | --- | --- | --- | --- | --- | --- | --- | --- | --- | --- | --- | --- | --- | --- | --- | --- | --- | --- | --- | --- | --- | --- | --- | --- | --- | --- | --- | --- | --- | --- | --- | --- | --- | --- | --- | --- | --- | --- | --- | --- | --- | --- | --- | --- | --- | --- | --- | --- | --- | --- | --- | --- | --- | --- | --- | --- | --- | --- | --- | --- | --- | --- | --- | --- | --- | --- | --- | --- | --- | --- | --- | --- | --- | --- | --- | --- | --- | --- | --- | --- | --- | --- | --- | --- | --- | --- | --- | --- | --- | --- | --- | --- | --- | --- | --- | --- | --- | --- | --- | --- | --- | --- | --- | --- | --- | --- | --- | --- | --- | --- | --- | --- | --- | --- | --- | --- | --- | --- | --- | --- | --- | --- | --- | --- | --- | --- | --- | --- | --- | --- | --- | --- | --- | --- | --- | --- | --- | --- | --- | --- | --- | --- | --- | --- | --- | --- | --- | --- | --- | --- | --- | --- | --- | --- | --- | --- | --- | --- | --- | --- | --- | --- | --- | --- | --- | --- | --- | --- | --- | --- | --- | --- | --- | --- | --- | --- | --- | --- | --- | --- | --- | --- | --- | --- | --- | --- | --- | --- | --- | --- | --- | --- | --- | --- | --- | --- | --- | --- | --- | --- | --- | --- | --- | --- | --- | --- | --- | --- | --- | --- | --- | --- | --- | --- | --- | --- | --- | --- | --- | --- | --- | --- | --- | --- | --- | --- | --- | --- | --- | --- | --- | --- | --- | --- | --- | --- | --- | --- | --- | --- | --- | --- | --- | --- | --- | --- | --- | --- | --- | --- | --- | --- | --- | --- | --- | --- | --- | --- | --- | --- | --- | --- | --- | --- | --- | --- | --- | --- | --- | --- | --- | --- | --- | --- | --- | --- | --- | --- | --- | --- | --- | --- | --- | --- | --- | --- | --- | --- | --- | --- | --- | --- | --- | --- | --- | --- | --- | --- | --- | --- | --- | --- | --- | --- | --- | --- | --- | --- | --- | --- | --- | --- | --- | --- | --- | --- | --- | --- | --- | --- | --- | --- | --- | --- | --- | --- | --- | --- | --- | --- | --- | --- | --- | --- | --- | --- | --- | --- | --- | --- | --- | --- | --- | --- | --- | --- | --- | --- | --- | --- | --- | --- | --- | --- | --- | --- | --- | --- | --- | --- | --- | --- | --- | --- | --- | --- | --- | --- | --- | --- | --- | --- | --- | --- | --- | --- | --- | --- | --- | --- | --- | --- | --- | --- | --- | --- | --- | --- | --- | --- | --- | --- | --- | --- | --- | --- | --- | --- | --- | --- | --- | --- | --- | --- | --- | --- | --- | --- | --- | --- | --- | --- | --- | --- | --- | --- | --- | --- | --- | --- | --- | --- | --- | --- | --- | --- | --- | --- | --- | --- | --- | --- | --- | --- | --- | --- | --- | --- | --- | --- | --- | --- | --- | --- | --- | --- | --- | --- | --- | --- | --- | --- | --- | --- | --- | --- | --- | --- | --- | --- | --- | --- | --- | --- | --- | --- | --- | --- | --- | --- | --- | --- | --- | --- | --- | --- | --- | --- | --- | --- | --- | --- | --- | --- | --- | --- | --- | --- | --- | --- | --- | --- | --- | --- | --- | --- | --- | --- | --- | --- | --- | --- | --- | --- | --- | --- | --- | --- | --- | --- | --- | --- | --- | --- | --- | --- | --- | --- | --- | --- | --- | --- | --- | --- | --- | --- | --- | --- | --- | --- | --- | --- | --- | --- | --- | --- | --- | --- | --- | --- | --- | --- | --- | --- | --- | --- | --- | --- | --- | --- | --- | --- | --- | --- | --- | --- | --- | --- | --- | --- | --- | --- | --- | --- | --- | --- | --- | --- | --- | --- | --- | --- | --- | --- | --- | --- | --- | --- | --- | --- | --- | --- | --- | --- | --- | --- | --- | --- | --- | --- | --- | --- | --- | --- | --- | --- | --- | --- | --- | --- | --- | --- | --- | --- | --- | --- | --- | --- | --- | --- | --- | --- | --- | --- | --- | --- | --- | --- | --- | --- | --- | --- | --- | --- | --- | --- | --- | --- | --- | --- | --- | --- | --- | --- | --- | --- | --- | --- | --- | --- | --- | --- | --- | --- | --- | --- | --- | --- | --- | --- | --- | --- | --- | --- | --- | --- | --- | --- | --- | --- | --- | --- | --- | --- | --- | --- | --- | --- | --- | --- | --- | --- | --- | --- | --- | --- | --- | --- | --- | --- | --- | --- | --- | --- | --- | --- | --- | --- | --- | --- | --- | --- | --- | --- | --- | --- | --- | --- | --- | --- | --- | --- | --- | --- | --- | --- | --- | --- | --- | --- | --- | --- | --- | --- | --- | --- | --- | --- | --- | --- | --- | --- | --- | --- | --- | --- | --- | --- | --- | --- | --- | --- | --- | --- | --- | --- | --- | --- | --- | --- | --- | --- | --- | --- | --- | --- | --- | --- | --- | --- | --- | --- | --- | --- | --- | --- | --- | --- | --- | --- | --- | --- | --- | --- | --- | --- | --- | --- | --- | --- | --- | --- | --- | --- | --- | --- | --- | --- | --- | --- | --- | --- | --- | --- | --- | --- | --- | --- | --- | --- | --- | --- | --- | --- | --- | --- | --- | --- | --- | --- | --- | --- | --- | --- | --- | --- | --- | --- | --- | --- | --- | --- | --- | --- | --- | --- | --- | --- | --- | --- | --- | --- | --- | --- | --- | --- | --- | --- | --- | --- | --- | --- | --- | --- | --- | --- | --- | --- | --- | --- | --- | --- | --- | --- | --- | --- | --- | --- | --- | --- | --- | --- | --- | --- | --- | --- | --- | --- | --- | --- | --- | --- | --- | --- | --- | --- | --- | --- | --- | --- | --- | --- | --- | --- | --- | --- | --- | --- | --- | --- | --- | --- | --- | --- | --- | --- | --- | --- | --- | --- | --- | --- | --- | --- | --- | --- | --- | --- | --- | --- | --- | --- | --- | --- | --- | --- | --- | --- | --- | --- | --- | --- | --- | --- | --- | --- | --- | --- | --- | --- | --- | --- | --- | --- | --- | --- | --- | --- | --- | --- | --- | --- | --- | --- | --- | --- | --- | --- | --- | --- | --- | --- | --- | --- | --- | --- | --- | --- | --- | --- | --- | --- | --- | --- | --- | --- | --- | --- | --- | --- | --- | --- | --- | --- | --- | --- | --- | --- | --- | --- | --- | --- | --- | --- | --- | --- | --- | --- | --- | --- | --- | --- | --- | --- | --- | --- | --- | --- | --- | --- | --- | --- | --- | --- | --- | --- | --- | --- | --- | --- | --- | --- | --- | --- | --- | --- | --- | --- | --- | --- | --- | --- | --- | --- | --- | --- | --- | --- | --- | --- | --- | --- | --- | --- | --- | --- | --- | --- | --- | --- | --- | --- | --- | --- | --- | --- | --- | --- | --- | --- | --- | --- | --- | --- | --- | --- | --- | --- | --- | --- | --- | --- | --- | --- | --- | --- | --- | --- | --- | --- | --- | --- | --- | --- | --- | --- | --- | --- | --- | --- | --- | --- | --- | --- | --- | --- | --- | --- | --- | --- | --- | --- | --- | --- | --- | --- | --- | --- | --- | --- | --- | --- | --- | --- | --- | --- | --- | --- | --- | --- | --- | --- | --- | --- | --- | --- | --- | --- | --- | --- | --- | --- | --- | --- | --- | --- | --- | --- | --- | --- | --- | --- | --- | --- | --- | --- | --- | --- | --- | --- | --- | --- | --- | --- | --- | --- | --- | --- | --- | --- | --- | --- | --- | --- | --- | --- | --- | --- | --- | --- | --- | --- | --- | --- | --- | --- | --- | --- | --- | --- | --- | --- | --- | --- | --- | --- | --- | --- | --- | --- | --- | --- |

**References**

1. Boessenkool S, McGlynn G, Epp LS, Taylor D, Pimentel M, Gizaw A, et al. Use of ancient sedimentary DNA as a novel conservation tool for high-altitude tropical biodiversity. Conserv Biol. 2014;28(2):446-55.

2. Angiosperm Phylogeny Group. An update of the Angiosperm Phylogeny Group classification for the orders and families of flowering plants: APG III. Botanical Journal of the Linnean Society. 2009;161(2):105-21.
