# Supplementary material for: DNA Metabarcoding Reveals Diet Overlap between the Endangered Walia Ibex and Domestic Goats - Implications for Conservation
Source: PLoS One. 2016 Jul 14;11(7):e0159133. doi: 10.1371/journal.pone.0159133 (PMC4945080; doi:10.1371/journal.pone.0159133)
Supplement: S2 Table — Listed MOTUs were detected more than 10 times in at least one of the two herbivore species. Occurrence refers to the number of sequence reads from the faecal samples of each species. Frequency refers to the number of samples where the MOTUs were detected. (DOCX) [file pone.0159133.s003.docx]

**Table S2. Diet** **molecular operational taxonomic units (MOTUs) detected in the samples.** Listed MOTUs were detected more than 10 times in at least one of the two herbivore species. Occurrence refers to the number of sequence reads from the faeces samples of each species. Frequency refers to the number of samples where the MOTUs were detected.

|  |  |  | **Walia ibex** | | **Goat** | |
| --- | --- | --- | --- | --- | --- | --- |
| **Plant family** | **Functional group** | **Plant taxon (MOTU)** | **Occurrence** | **Frequency** | **Occurrence** | **Frequency** |
| Rosaceae | Forb | *Alchemilla* sp. | 36438 | 18 | 19645 | 8 |
|  | Shrub/tree | *Rosa abyssinica* | 504 | 7 | 22742 | 12 |
| Hypericaceae | Shrub/tree | *Hypericum revolutum* | 24967 | 20 | 7066 | 14 |
| Ericaceae | Shrub/tree | *Erica arborea* | 21237 | 19 | 58679 | 15 |
| Asteraceae | Shrub/tree | *Helichrysum* sp. | 22796 | 14 | 0 | 0 |
|  |  | *Inula arbuscula* | 9356 | 7 | 0 | 0 |
|  |  | *Helichrysum horridum* | 1768 | 7 | 144 | 7 |
|  |  | *Vernonia* sp. | 31 | 1 | 407 | 9 |
|  | Forb | *Carduus* sp. | 1882 | 12 | 333 | 4 |
|  |  | *Dichrocephala* sp. | 14 | 1 | 18 | 1 |
|  |  | *Haplocarpha* sp. | 83 | 2 | 0 | 0 |
|  |  | *Senecio* sp. | 228 | 5 | 0 | 0 |
|  | - | Astereae sp. | 673 | 12 | 77 | 2 |
|  | - | Asteraceae sp. | 245 | 6 | 2754 | 15 |
| Polygalaceae | Forb | *Rumex* sp. | 2516 | 14 | 50860 | 16 |
| Crassulaceae | Shrub/tree | *Aeonium leucoblepharum* | 4948 | 7 | 248 | 2 |
| Urticaceae | Forb | *Parietaria debilis* | 2340 | 2 | 0 | 0 |
| Campanulaceae | Shrub/tree | *Lobelia rhynchopetalum* | 1734 | 12 | 172 | 3 |
| Lamiaceae | Forb | *Thymus schimperi* | 1811 | 5 | 2857 | 9 |
|  |  | Mentheae sp. | 86 | 2 | 237 | 5 |
|  |  | *Nepeta azurea* | 802 | 7 | 0 | 0 |
|  | Shrub/tree | *Satureja* sp. | 71 | 4 | 77 | 4 |
| - | - | Lamiales sp. | 0 | 0 | 2509 | 7 |
| Rubiaceae | Forb | *Galium* sp. | 1068 | 7 | 183 | 5 |
|  |  | *Veronica* sp. | 15 | 1 | 58 | 3 |
|  | Shrub/tree | *Anthospermum pachyrrhizum* | 69 | 1 | 79 | 3 |
|  |  | *Galiniera saxifraga* | 0 | 0 | 29 | 2 |
| Onagraceae | Forb | *Epilobium stereophyllum* | 952 | 11 | 41 | 2 |
| Dryopteridaceae | Forb | *Dryopteris schimperiana* | 854 | 9 | 25 | 2 |
| Poaceae | Graminoid | *Festuca* sp. | 549 | 12 | 368 | 7 |
|  |  | *Andropogon* sp. | 30 | 1 | 134 | 5 |
|  |  | PACMAD clade | 0 | 0 | 80 | 3 |
|  |  | *Pennisetum* sp. | 0 | 0 | 40 | 2 |
|  |  | Pooideae | 0 | 0 | 148 | 6 |
|  |  | *Rytidosperma subulata* | 0 | 0 | 192 | 2 |
| Primulaceae | Shrub/tree | *Myrsine* sp. | 382 | 2 | 3430 | 12 |
| Celastraceae | Shrub/tree | *Maytenus* sp. | 383 | 4 | 92 | 4 |
| Ranunculaceae | Shrub/tree | *Clematis* sp. | 350 | 6 | 161 | 3 |
|  | Forb | *Ranunculus* sp. | 130 | 2 | 0 | 0 |
| Solanaceae | Shrub/tree | *Discopodium penninervium* | 33 | 1 | 28 | 1 |
|  | - | *Solanum* sp. | 239 | 2 | 190 | 6 |
| Saxifragaceae | Forb | *Saxifraga hederifolia* | 215 | 2 | 0 | 0 |
| Scrophulariaceae | Forb | *Hebenstretia angolensis* | 208 | 3 | 0 | 0 |
| Brassicaceae | Forb | Brassicaceae | 138 | 5 | 32 | 2 |
| Apiaceae | Forb | *Haplosciadium abyssinicum* | 105 | 4 | 0 | 0 |
|  |  | *Heracleum abyssinicum* | 12 | 1 | 48 | 3 |
|  |  | *Agrocharis* sp. | 0 | 0 | 66 | 3 |
| Xanthorrhoeaceae | Forb | *Kniphofia* sp. | 74 | 2 | 68 | 3 |
| Olaceae | Shrub/tree | *Olea* sp. | 42 | 2 | 5455 | 12 |
|  | Shrub/tree | *Jasminum* sp. | 0 | 0 | 272 | 9 |
| Myricaceae | Shrub/tree | *Myrica salicifolia* | 38 | 1 | 1093 | 6 |
| Fabaceae | Forb | *Trifolium* sp. | 11 | 1 | 71 | 4 |
| Boraginaceae | Forb | *Cynoglossum* sp. | 10 | 1 | 10 | 1 |
| Caprifoliaceae | Forb | *Scabiosa columbaria* | 0 | 0 | 90 | 3 |
